# Systematic review and meta-analysis of whether cesarean section contributes to the incidence of allergic diseases in children: A protocol for systematic review and meta analysis

**DOI:** 10.1097/MD.0000000000018394

**Published:** 2019-12-27

**Authors:** Li Gu, Weijian Zhang, Wenhao Yang, Hanmin Liu

**Affiliations:** a Department of Pediatric Pneumology; b Key Laboratory of Birth Defects and Related Diseases of Women and Children (Sichuan University), Ministry of Education, West China Second University Hospital, Sichuan University, Chengdu, China.

**Keywords:** allergy, caesarean section, children, meta-analysis

## Abstract

**Background:**

As abundant evidence shows the composition of gut flora in children born by caesarean section is different from that of vaginal delivery children, studies on whether caesarean section would increase the offspring's risk of developing allergic disease attract extensive attention. However, the results of different researches are inconsistent. Therefore we conduct a systematic review and meta-analysis to explore the relationship between caesarean section and childhood allergic disease.

**Methods:**

The protocol followed Preferred Reporting Items for Systematic Reviews and Meta-Analyses Protocols. Cohort studies for investigating the relationship between caesarean section and the risk of childhood allergic disease will be searched in 4 main databases (PubMed, EMBASE, the Cochrane Library, and the web of science). In addition, a manual search of the references of relevant published studies will also be considered. Four common allergic outcomes will be included: asthma, allergic rhinitis, food allergy, and atopic dermatitis. Studies selection, data extraction, and risk of bias assessment will be conducted by 2 independent reviewers. The primary outcome is the incidence of 4 allergic diseases.

**Results:**

The results will provide useful information on whether caesarean section contributes to the increase of allergic disease in children.

**Conclusion:**

The findings of this study will be published in a peer-reviewed journal.

**PROSPERO registration number:**

CRD42019135196.

## 1 Introduction

Over the past decade, the rate of caesarean section (CS) has continued to trend upward.^[1]^ Based on the World Health Organization global survey, the CS rate varies widely across the geographical regions, with country-level rate ranging from less than 10% to more than 50%.[^2^,^3^,^4^] Although evidence has shown CS can reduce risks of maternal and perinatal mortality and morbidity,[^5^,^6^] the long-term risks and benefits of CS, especially those without medical indications, remain unclear.[^7^,^8^]

Allergic disease is a common chronic disease, and could come on at any age in childhood. Reports on the correlation between CS and the offspring's risk of developing allergic disease are of current interest due to the high rate of CS in many countries, but the results are inconsistent.[^9^,^10^,^11^] Evidence shows that the composition of microbiome in the fetal and neonatal period plays a key role in the development of the immune system, and CS babies have a different gut flora in comparison with vaginal delivery babies, which is considered to be 1 main reason why CS may increase the risk of later development of allergic disease.[^12^,^13^,^14^]

Whether CS could increase the risk of childhood allergic disease is still a controversial topic, therefore we conducted a review and meta-analyses of epidemiological studies of delivery by CS and risk of allergic outcomes to explore this relationship.

## 2 Methods

### 2.1 Registration

This protocol has been registered in the PROSPERO and the registration number is CRD42019135196. This meta-analysis and systematic review will be based on the guideline of the Cochrane Handbook for Systematic Reviews of Interventions^[15]^ and the PRISMA (Preferred Reporting Items for Systematic Reviews andMeta-Analyses) statement.^[16]^ The software RevMan 5.2 and STATA version 14.0 (College Station, TX) will be used to construct the meta-analysis. Ethical approval are not required because there is no direct involvement of human.

### 2.2 Type of participants

Participants who must be diagnosed with 1 or more allergic diseases including asthma, allergic rhinitis, atopic dermatitis, and food allergy by specialist will be included. Participants must be less than 18 years old. There are no restrictions on race and gender.

### 2.3 Type of exposed group and control group

Participants delivered by CS will be considered as the exposed group. CS is divided into elective and emergency CS. Elective CS is a planned delivery performed on pregnant woman, which is often conducted before the onset of labor because of either maternal or fetal indications. Emergency CS is defined as CS in an obstetric emergency generally after the onset of labor. The control group is defined as those delivered by vaginal delivery.

### 2.4 Type of outcome measures

Incidence of asthma, allergic rhinitis, allergic dermatitis, and food allergy is the only outcome. The definition of the above 4 allergic diseases is based on physician's diagnosis.

### 2.5 Type of studies

Cohort study in English with a sample size of more than 1000 will be included without restriction of publication type. In addition, the follow-up time must be more than 1 year.

### 2.6 Search methods for the identification of studies

We will search the following electronic bibliographic databases with no language restrictions: PubMed, EMBASE, the Cochrane Library. Studies published between January 1990 and the date the searches are run will be sought. The searches will be re-run just before the final analyses and further studies retrieved for inclusion. In addition, We will search for qualified references through the list of references in other literatures, and try to contact the authors for the full text when it is not available. Table 1 shows the full list of search terms to be used.

**Table 1:**
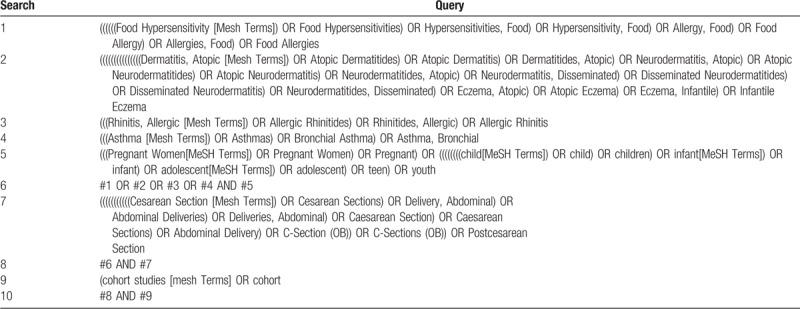
Preliminary search strategy in PubMed.

## 3 Data collection and analysis

### 3.1 Selection of studies

**Figure 1:**
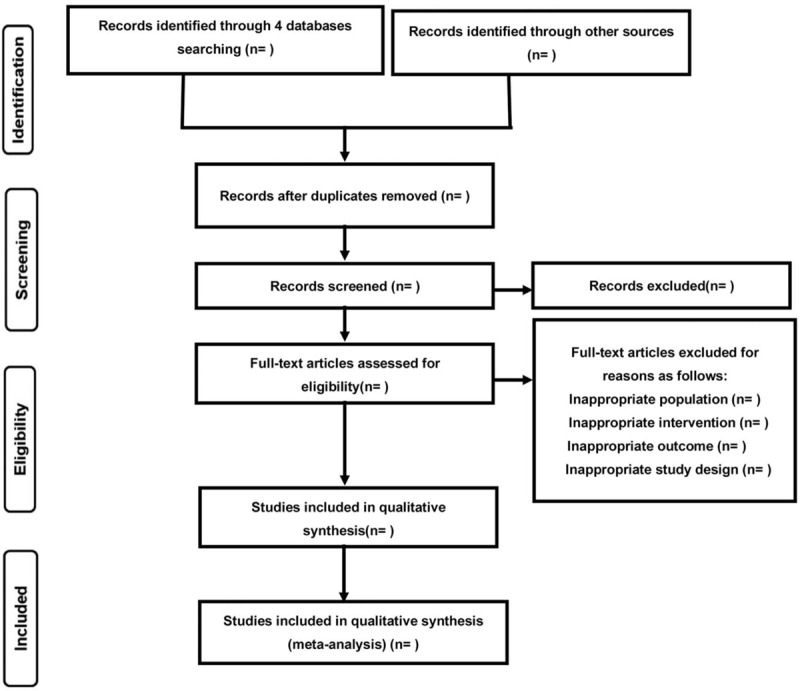
Flow diagram of study selection.

Two reviewers will perform the studies selection at the same time independently. First, duplicate papers will be removed, and then the unqualified literatures will be deleted by scanning the title and abstract. Finally, the full text of remaining studies will be screened for further exclusion. Excluded studies will be listed in a table with reasons. If any disagreement occurs, a decision will be made through discussion or consultation with a third author. Details of the study selection procedure are shown in Figure 1.

### 3.2 Data extraction

Before data extraction, a standard form containing study characteristics, patient characteristics, data needed for quality assessment, and outcomes will be created. Two reviewers will conduct data extraction independently according to this standard, and will check the data with each other when data extraction finished. During the process, any disagreements will be resolved by negotiation.

### 3.3 Quality assessment of included studies

Newcastle Ottawa Scale recommended by Cochrane Collaboration's tool for non-randomized controlled trials will be employed to assess the reporting quality of included original studies. This scale consists of 8 items evaluating the quality of observational cohort studies in terms of selection, comparability, and outcome. Differences in opinion will be resolved by discussion or consultation with a third author.

## 4 Data synthesis and statistical analysis

### 4.1 Data synthesis

Revman 5.2 software and TATA version 14.0 (College Station, TX) will be used to conduct the meta-analysis. For dichotomous data, the incidence of asthma, AR, AD, and FA, will be reported as risk ratios (RRs) with their 95% confidence intervals (CIs). *P* < .05 is considered to be statistically significant.

### 4.2 Assessment of heterogeneity

Heterogeneity will be assessed by the *I* ^2^ test. The value of *I* ^2^ ranges from 0% to100%. with 0% to 40% indicating no important heterogeneity, 40% to 60% indicating moderate heterogeneity, 60% to 90% indicating substantial heterogeneity, and >90% indicating considerable heterogeneity. If *I* ^2^ ≥ 50%, the reasons for the high heterogeneity will be searched and a random-effects model for data analysis will be used. In addition, on the basis of the source of heterogeneity, subgroup analysis may be conducted.

### 4.3 Assessment of publication bias

A funnel plot will be used to evaluate publication bias if more than 10 studies are included. RR from each study is plotted against their variance. Asymmetrical appearance of the plot indicates the presence of publication bias. Egger test will be used to test the asymmetry of the funnel plot. The results will be calcified based on the Cochrane Handbook for Systematic Reviews of Interventions.

### 4.4 Sensitivity analysis

The sensitivity analysis will be performed to assess whether the sample size and missing data impact the results of the review. If there are adequate studies (no less than 3 studies), we will conduct a sensitivity analysis to check the robustness of conclusions and assess the impact of methodological quality.

## 5 Discussion

Allergic disease in children is constantly increasing and there is evidence shows children born by CS have a different gut flora, which has been considered to postpone immunological immaturity and therefore increase the risk of allergic disease. In addition, CS is a well-recognized cause of transient tachypnea of the newborn and respiratory distress syndrome, which are associated with an increased risk of pediatric asthma.[^17^,^18^,^19^] Despite these theories, previous studies have reported inconsistent results. We present a protocol of a systematic review and meta-analysis to determine the relationship between CS and childhood allergic disease. The conclusions drawn from this review will benefit pregnant women and physicians seeking appropriate mode of delivery.

## Author contributions

LG come up with conception of this study and drafted the preliminary version of this protocol. LG and WJZ will perform the study search, study selection, data extraction and the assessment of publication bias. LG, WJZ and WHY will complete the data analysis. HML will help to solve any disagreement and ensure the quality of this study. All authors have approved the publication of the protocol.
